# The DegU Orphan Response Regulator Contributes to Heat Stress Resistance in *Listeria monocytogenes*

**DOI:** 10.3389/fcimb.2021.761335

**Published:** 2021-12-13

**Authors:** Changyong Cheng, Feng Liu, Haobo Jin, Xiangfei Xu, Jiali Xu, Simin Deng, Jing Xia, Yue Han, Lei Lei, Xian Zhang, Houhui Song

**Affiliations:** College of Animal Science and Technology & College of Veterinary Medicine of Zhejiang Agriculture and Forestry University, Key Laboratory of Applied Technology on Green-Eco-Healthy Animal Husbandry of Zhejiang Province, China-Australia Joint Laboratory for Animal Health Big Data Analytics, Zhejiang Provincial Engineering Laboratory for Animal Health Inspection & Internet Technology, Hangzhou, China

**Keywords:** *Listeria monocytogenes*, orphan response regulator, DegU, heat resistance, heat-shock proteins

## Abstract

*Listeria monocytogenes* is more heat-resistant than most other non-spore-forming foodborne pathogens, posing a *severe* threat to food safety and human health, particularly during chilled food processing. The DegU orphan response regulator is known to control heat resistance in *L. monocytogenes*; however, the underlying regulatory mechanism is poorly understood. Here, we show that DegU contributes to *L. monocytogenes* exponential growth under mild heat-shock stress. We further demonstrate that DegU directly senses heat stress through autoregulation and upregulates the *hrcA-grpE-dnaK-dnaJ* operon, leading to increased production of heat-shock proteins. We also show that DegU can directly regulate the expression of the *hrcA-grpE-dnaK-dnaJ* operon. In conclusion, our results shed light on the regulatory mechanisms underlying how DegU directly activates the *hrcA-grpE-dnaK-dnaJ* operon, thereby regulating heat resistance in *L. monocytogenes*.

## Introduction

*Listeria monocytogenes* is a facultative intracellular Gram-positive bacterium that causes listeriosis, which is associated with a mortality rate of 20%-30% in immunocompromised individuals (de Noordhout et al., 2014; Pasechnek et al., 2020). This bacterial pathogen is widespread in the environment and can survive under a wide range of stress conditions, such as low and high temperatures, dryness, low pH, and high salinity, which allow it to persist in food manufacturing sites for several years (Ming and Daeschel, 1993; Keto-Timonen et al., 2007; Camargo et al., 2017). *L*. *monocytogenes* can reportedly grow under temperatures as high as 45°C and is more heat resistant than many other non-spore-forming foodborne pathogens (Lin et al., 2012; Pontinen et al., 2017; Ballom et al., 2020). Although the general heat stress properties of *L. monocytogenes* have been described (Nair et al., 2000; van der Veen et al., 2007; Soni et al., 2011), further investigations are required to reveal the response mechanisms triggered by heat stress in the pathogen.

Pathogenic bacteria rely on two-component systems (TCSs) to sense chemical and physical changes in the environment and respond accordingly (Lemmer et al., 2020; Salvail and Groisman, 2020). Canonical TCSs comprise a signal-sensing histidine kinase and a cytoplasmic response regulator. In these systems, the histidine kinase undergoes autophosphorylation under certain stimuli and activates a cognate response regulator *via* the transfer of the phosphoryl group (Murret-Labarthe et al., 2020; Rapun-Araiz et al., 2020; Tiwari et al., 2020). Genome-wide analysis has revealed that *L*. *monocytogenes* encodes 15 paired two-component systems and one orphan response regulator (Williams et al., 2005).

The DegS/DegU two-component system of *B. subtilis* was one of the first described in Gram-positive bacteria (Tokunaga et al., 1994; Mader et al., 2002; Cairns et al., 2015); however, unlike *B. subtilis*, *L*. *monocytogenes* expresses only DegU, the cognate response regulator, and not DegS, the sensor histidine kinase. Hence, DegU is considered an orphan response regulator in this bacterium (Mauder et al., 2008). Previous studies have shown that DegU is a pleiotropic regulator in *L. monocytogenes*, and is required for biofilm formation, chemotaxis, motility, and growth at high temperatures (Gueriri et al., 2008a; Gueriri et al., 2008b; Mauder et al., 2008). Although DegU has been reported to play a crucial role in resistance to heat stress in *L. monocytogenes*, how DegU allows it to respond rapidly to rising temperatures is unknown. Here, the principal objective of this study was to investigate the mechanism underlying the DegU-mediated heat-stress tolerance in this bacterium.

## Materials and Methods

### Bacterial Strains and Primers

*L. monocytogenes* strain EGD-e was used as the reference strain. All *Escherichia coli* strains were grown at 37°C in LB medium (Oxoid Ltd, Basingstoke, United Kingdom), and all *L. monocytogenes* strains were grown at 37°C in BHI broth (Oxoid Ltd). *E. coli* DH5α was used for transformation, and *E. coli* BL21 was used for protein expression. When needed, the following antibiotics were added to *E. coli* or *L. monocytogenes* cultures: 100 µg/mL ampicillin, 10 µg/mL chloramphenicol, or 50 µg/mL kanamycin. Primers were purchased from Tsingke (Tsingke Biotechnology Co., Ltd, Hangzhou, China), and the sequences are shown in **Table S1**.

### Construction of Mutant and Complementary Strains

The *ΔdegU* mutant was constructed by a two-step allelic exchange procedure using the pKSV7 shuttle plasmid as previously described (Cheng et al., 2021). The *degU* complementation strains were generated using the integrative plasmid pIMK2 as previously described (Zhang et al., 2020). The targeted *degU* gene was cloned into pIMK2 *via* a one-step cloning method and then electroporated into competent *L. monocytogenes* cells. The mutant and complement strains were verified by PCR and DNA sequencing.

### β-Galactosidase Assay

A *degU*-*lacZ* fusions containing the promoter region of *degU* and the *lacZ* gene, was cloned into the Sac I and BamH I sites of the plasmid pIMK2. Then, the recombinant plasmid was introduced into the wild-type EGD-e and the Δ*degU* mutant strain. All strains were grown overnight in BHI broth at 37°C, diluted 1:1,000 in fresh BHI broth, and grown at 43°C to an OD600 of 0.6. The collected culture was assayed for β-Galactosidase activity using a β-galactosidase (β-GAL) Activity Assay Kit(Micromethod; Sangon Biotech) according to the manufacturer’s specification.

### Real-Time Quantitative Reverse Transcription-PCR (RT-qPCR)

The wild-type, *ΔdegU* mutant, and complement strains were grown in BHI broth overnight, diluted 1:1,000 in fresh BHI broth, and incubated at 43°C to an OD_600_ of 0.6. Total bacterial RNA was extracted using the Bacteria Total RNA Isolation Kit (Sangon Biotech, China) and reverse-transcribed into cDNA using reverse transcriptase from TOYOBO. According to the manufacturer**’**s specifications, real-time qPCR was performed using an Mx3000P qPCR system (Stratagene-Agilent) and SYBR qPCR Mix (TOYOBO). All expression results were normalized relative to the housekeeping gene *rpoB*. Relative transcription levels were determined using the 2^−ΔΔCt^ method. RT-PCR was performed as previously described (Liu et al., 2018). The PCR products were electrophoresed on a 1% agarose gel and photographed using a SmartView Pro 2400 system (Major Science, USA).

### Expression and Purification of Recombinant Proteins

The *degU* gene was amplified from genomic DNA of L. monocytogenes EGD-e with the primer pair PdegU-F and PdegU-R and cloned into the Nde I and Xho I sites of the expression vector pET30a. Then, the recombinant plasmid pET30a-degU was transformed into *E. coli* BL21 to express His6-DegU protein. The expression was induced by isopropyl-β-D-thiogalactopyranoside (0.5 mM) at 16°C for 5 h and purified by Ni-nitrilotriacetic acid (Ni-NTA) resin affinity chromatography. The purified proteins were confirmed by running the SDS-PAGE and stored at -80°C until use.

### Electrophoretic Mobility Shift Assay (EMSA)

The recombinant protein DegU was obtained and phosphorylated according to previously described procedures (Goodman et al., 2020). DNA probes were purified with a Gel Extraction Kit (TIANGEN, China) and were labeled using the Biotin Labeling Kit for the EMSA (Beyotime, China). EMSA was performed with the Chemiluminescent EMSA Kit (Beyotime). Samples were analyzed by 4% non-denaturing polyacrylamide gel electrophoresis in 0.5 × TBE buffer. The gel was then transferred to a nylon membrane (Beyotime) followed by UV crosslinking. The bands were detected using the Chemiluminescent EMSA Kit (Beyotime).

### DNase I Footprinting Assay

DNase I footprinting experiments were carried out as previously described (Li et al., 2018). To prepare the fluorescent FAM-labeled probes, the promoter region of *hrcA* was PCR amplified using a 2× KOD One PCR Master Mix (TOYOBO) from the plasmid T-*hcrA* using primers containing 6-FAM at the 5′ end. The labeled probes (300 ng) were then mixed with purified DegU in a 40-µL reaction volume at 25°C for 30 min. Subsequently, 0.015 units of DNase I (Promega) and the reaction buffer were added, followed by incubation for 1 min at 37°C. The reaction was terminated by adding 140 µL of DNase I stop solution. Digested DNA samples were extracted with phenol-chloroform, and pellets containing DNA were resuspended in 30 µL of water. The results were analyzed using Peak Scanner software v1.0 (Applied Biosystems).

### Statistical Analysis

Data were analyzed using GraphPad Prism version 5.0 (GraphPad Software, La Jolla, CA, USA) using two-tailed Student’s *t*-tests and are presented as means ± SD.

## Results

### The Role of the Orphan Response Regulator DegU in Heat Resistance

**Figure 1:**
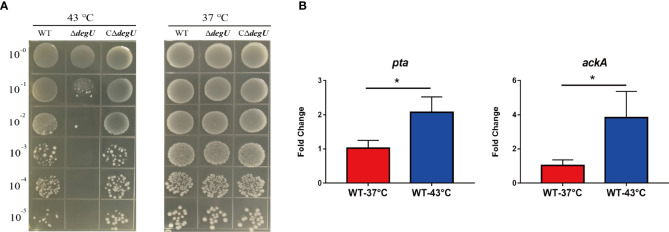
DegU modulates *Listeria monocytogenes* responses to heat stress. **(A)** Bacterial growth under heat stress. The wild-type (WT) EGD-e and the ΔdegU mutant *L. monocytogenes* were spotted on BHI agar plates using 10-fold serial dilutions and incubated at 37°C and 43°C. **(B)** The relative expression levels of the *pta* and *ackA* genes in response to heat stress. WT *L. monocytogenes* EGD-e cells were grown at 43°C. Data are presented as the means ± S.D. of three independent experiments. *p < 0.05.

To verify whether DegU plays an important role in the heat tolerance of *L. monocytogenes*, we generated the in-frame deletion mutant strain *ΔdegU* and the complemented strain C*ΔdegU*. When exposed to heat stress (43°C), the mutant strain *ΔdegU* showed a significant growth defect on BHI agar plates compared with the wild-type EGD-e and CΔ*degU* strains (**Figure 1A**). The promoter activity of P_degU_-*lacZ* in the WT and Δ*degU* strains had no obvious change under heat stress (**Figure S3**), which indicated that DegU protein level was not elevated under heat stress. Previous studies have demonstrated that the Pta-AckA pathway plays a role in DegU protein phosphorylation (Gueriri et al., 2008a). The RT-qPCR results showed that the mRNA level of the *pta* and *ackA* genes, which are responsible for DegU activation, were significantly elevated under heat stress in the WT strain (**Figure 1B**). Collectively, these results strongly indicated that DegU contributes to the heat resistance of *L. monocytogenes*.

### DegU Transcriptionally Regulates the Expression of the *hrcA-grpE-dnaK-dnaJ* Operon Under Heat Stress

**Figure 2:**
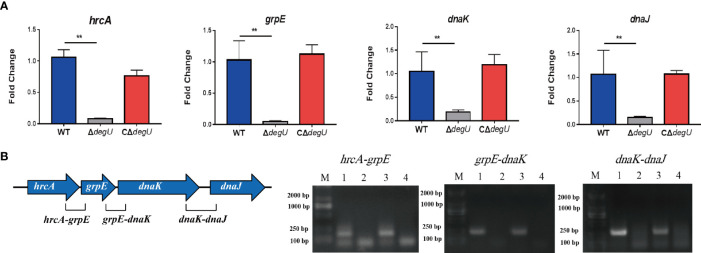
hrcA-dnaJ transcription is activated by DegU. **(A)** The relative mRNA levels of hrcA, grpE, dnaK, and dnaJ as determined by RT-qPCR in WT, *ΔdegU*, and C*ΔdegU* strains at 43°C. **(B)** Schematic diagram showing the gene order. Co-expression results confirmed that the hrcA, grpE, dnaK, and dnaJ genes form an operon under heat-stress conditions. Lane 1, cDNA; lane 2, total RNA; lane 3, genomic DNA; and lane 4, no-template control. Data are presented as the means ± S.D. of three independent experiments. **p < 0.01.

Many heat-shock-related genes have been previously identified, including *hrcA*, *grpE*, *dnaK dnaJ*, *htpX*, and *groEL* (Diamant and Goloubinoff, 1998; Arsene et al., 2000; van der Veen and Abee, 2010). In this study, we examined the link between DegU and the transcript levels of these genes using RT-qPCR. Under heat stress, the transcript levels of the *hrcA*, *grpE*, *dnaK*, and *dnaJ* genes were significantly lower in the *ΔdegU* mutant strain than in the wild-type EGD-e and complemented strains (**Figure 2A**), and the *htpX* and *groEL* genes were not changed (data not show). These four genes are adjacent *to* the *L. monocytogenes* chromosome (**Figure 2B**). To verify if they are co-transcribed as a polycistronic mRNA under heat stress, we performed RT-PCR across the *hrcA*-*grpE*, *grpE*-*dnaK*, and *dnaK*-*dnaJ* junctions. The results confirmed that the *hrcA*, *grpE*, *dnaK*, and *dnaJ* genes comprise an operon (**Figure 2B**). However, it should be recognized that RT-PCR is not sufficient to conclude that these genes constitute a complete operon, which may be short overlapping transcripts generated from internal promoters and terminators. These findings indicated that DegU is essential for the transcriptional regulation of the *hrcA*-*grpE*-*dnaK*-*dnaJ* operon.

### DegU Binds Specifically to the *hrcA* Promoter Region

**Figure 3:**
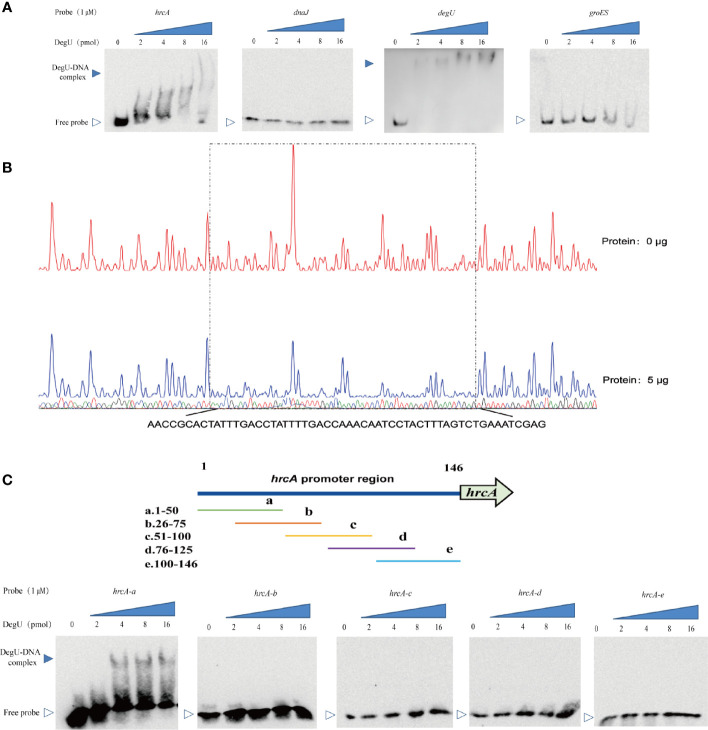
Identification of DegU binding sites in the *hrcA* promoter. **(A)** Electrophoretic mobility shift assay (EMSA) analysis of the direct binding of DegU-P to the *hrcA*, *dnaJ*, *degU* (positive control), and *groES* (negative control) promoter regions. **(B)** DNase I footprinting assay analysis of DegU-P binding to the *hrcA* promoter. FAM-labeled *hrcA* probes (300 ng) were used for the binding reactions in the absence (upper panel) or presence of 5 µg (lower panel) of DegU. The nucleotide sequences protected by DegU binding are shown below the electropherogram. **(C)** Electrophoretic mobility shift assay (EMSA) analysis of the binding of DegU-P to various truncated forms of the *hrcA* promoter. A total of six probes (left panel) were used to identify the DegU binding site in the *hrcA* promoter region by EMSA (right panel). Each experiment was performed three times, and the representative results were shown.

To further investigate the mechanism involved in the DegU-mediated regulation of the *hrcA*-*grpE*-*dnaK*-*dnaJ* operon, we determined the binding site for DegU in the promoter regions of *hrcA* and *dnaJ* using EMSA, with the *degU* and *groES* promoters respectively serving as a positive or negative control for DegU binding. Marked band shifts were observed with the *hrcA* promoter region but not with that of *dnaJ* (**Figure 3A**). These EMSA data showed that DegU bound to the promoter region of *hrcA* but not to that of *dnaJ*. To map the precise binding sequence of DegU, a DNase I footprinting assay was performed using FAM-labeled probes. As shown in **Figure 3B**, a 56-bp sequence (3′-AACCGCACTATTTGACCTATTTTGACCAAACAATCCTACTTTAGTCTGAAATCGAG-5′) appeared to be protected from DNase I digestion by DegU binding. To identify the minimum sequence required for DegU binding, the fragment of the *hrcA* promoter region used for EMSA was divided into segments so that the specific binding site was confined within the remaining 50 bp (**Figure 3C**).

### The Characteristic of the DegU Binding Site

**Figure 4:**
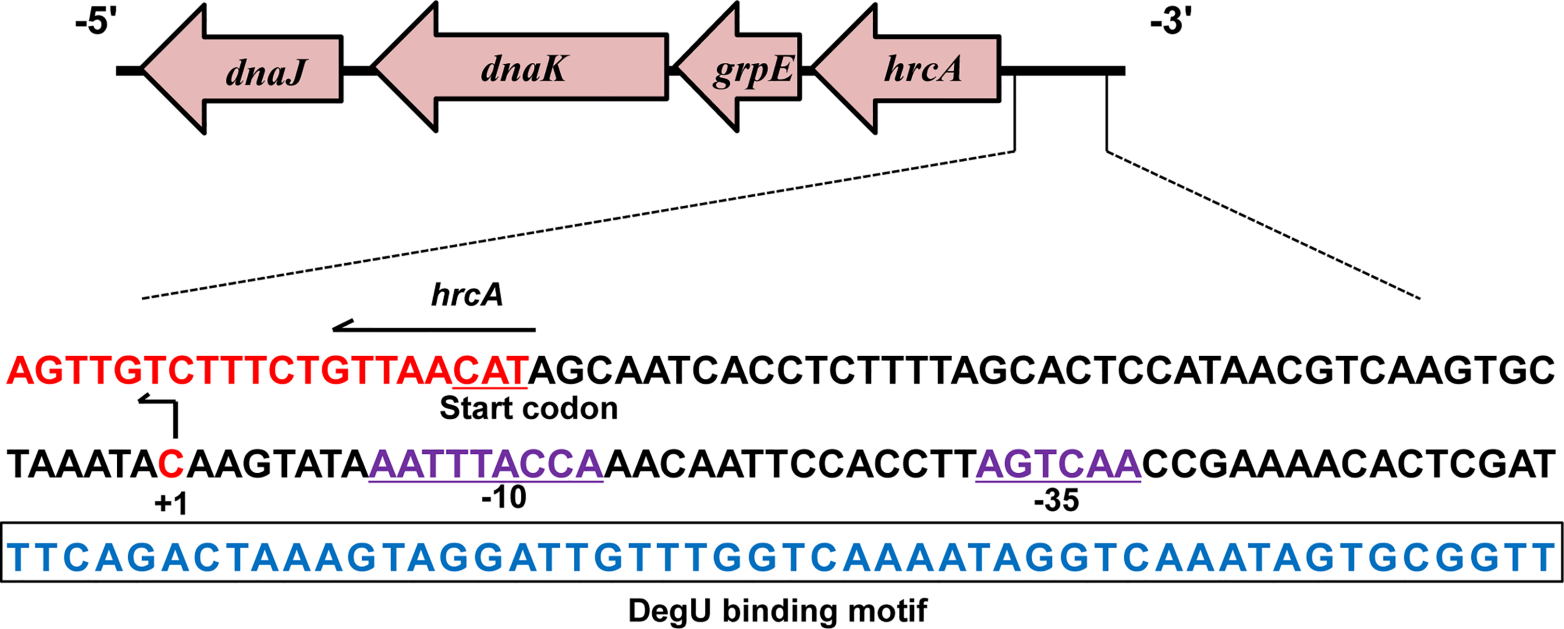
Characterizing the DegU Binding Site. DNA sequences of the hrcA promoter region. The DegU-binding site is shown in blue nucleotides boxed in black. The -35 and -10 regions are underlined and shown in purple. The TSS is denoted by +1 (bent arrow). The start codon of hrcA is shown in red. In panels A, experiment was performed at least three times and representative result is shown.

According to previous studies, the TSS (designated as +1) of *hrcA* was found to be located 45 bp upstream of its start codon and designated as C (**Figure 4**) (Wurtzel et al., 2012). In addition, analysis of the *hrcA* promoter region revealed a putative −10 AATTTACCA box and a putative −35 AGTCAA box respectively located at 8 bp and 31 bp downstream of the TSS (**Figure 4**). Furthermore, the specific DegU binding sequence was mapped from 52 to 101 bp upstream from the *hrcA* TSS (**Figure 4**).

## Discussion

The DegS/DegU system is a pleiotropic TCS of *B. subtilis* involved in controlling many biological processes, such as chemotaxis, motility, and degradative enzyme production (Gupta and Rao, 2014). In *L*. *monocytogenes*, DegU is considered an orphan response regulator as this bacterium lacks DegS, the sensor histidine kinase (Gueriri et al., 2008a). Studies have previously shown that DegU is required for *L. monocytogenes* to grow in RPMI 1640 synthetic medium and BHI broth at 44°C (Gueriri et al., 2008b). In our study, experimental mutation of the *degU* gene in this pathogen inhibited its growth on BHI agar at 43°C, suggesting that DegU plays a crucial role in heat resistance in *L. monocytogenes*.

In *L. monocytogenes* LM1009, the deletion of the *pta* and *ackA* genes completely blocked acetyl phosphate synthesis, suggesting that Pta and AckA are essential for the synthesis of acetyl phosphate which plays an important role in modulating DegU activity in *L. monocytogenes* (Gueriri et al., 2008a). So, the mRNA level of the *pta* and *ackA* genes elevated under heat stress could enhance DegU activity. That the *degU* mutant could not respond to heat stress suggested that DegU is essential for inducing the transcription of heat-shock proteins in *L. monocytogenes*. To elucidate the underlying regulatory mechanisms, several differentially expressed heat-shock-related genes were selected for RT-qPCR analysis of the transcriptional changes induced by heat stress (Kornitzer et al., 1991; Hanawa et al., 2000; Cardoso et al., 2010; Somolinos et al., 2010). The results suggested that heat-shock-related genes such as *hrcA*, *grpE*, *dnaK*, and *dnaJ*, well-known to play important roles in response to heat shock, were under the control of DegU in *L. monocytogenes*.

As the fact is that the class I heat-shock response is activated under heat shock and is essential for prokaryotic cells surviving in environmental stresses. Previous studies have shown that HrcA is a transcription repressor for the class I heat-shock response; GrpE, DnaJ, and DnaK are the class I heat-shock response chaperone proteins (van der Veen and Abee, 2010). DnaK can bind denatured proteins and assists the refolding of denatured polypeptides into active proteins (Hartl, 1996; Pierpaoli et al., 1997). DnaJ and GrpE can increase the rate of protein folding and release from DnaK through the transfer of non-native proteins to DnaK (Liberek et al., 1991).

As previously reported, the *hrcA*-*grpE*-*dnaK*-*dnaJ* operon can be transcribed from various sites (Hanawa et al., 2000). Sequence analysis led to the identification of the promoter sequence and two transcriptional initiation sites, one upstream of *hrcA* and the other upstream of *dnaJ*, which corresponded to the independent expression of the *dnaJ* gene (Hanawa et al., 2000). Interestingly, RT-PCR analysis showed that *hrcA*, *grpE, dnaK*, and *dnaJ* were co-transcribed as a single transcript from the transcriptional initiation site of the *hrcA* gene under heat stress conditions. EMSA and DNase I footprinting indicated that DegU directly interacted with a 50-bp sequence in the *hrcA* promoter region but did not bind to the *dnaJ* promoter.

**Figure 5:**
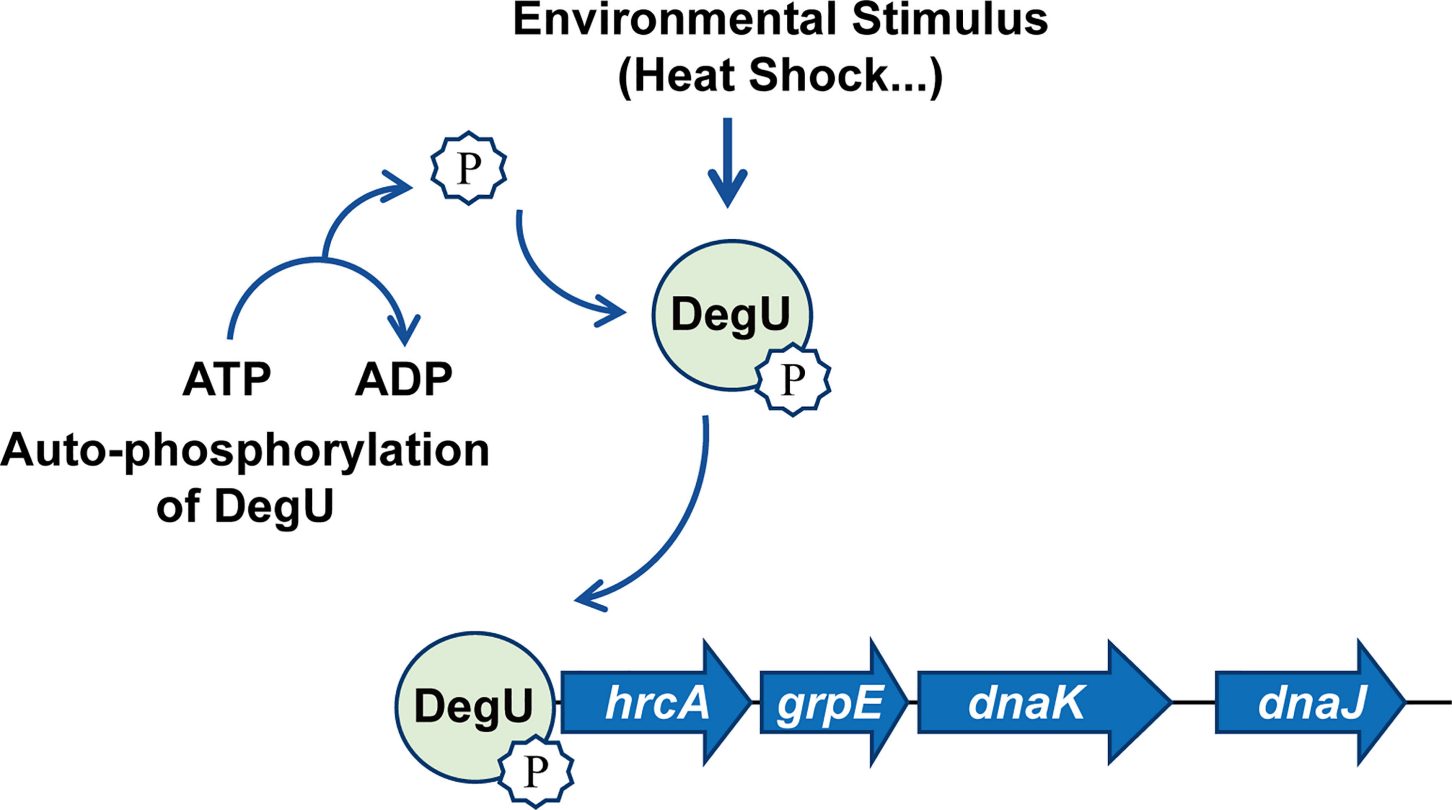
Model illustrating that DegU mediates heat resistance in *Listeria monocytogenes*. Under heat stress, DegU responds by undergoing autophosphorylation and directly activating the transcription of the *hrcA* heat-shock operon.

In conclusion, for the first time, we have revealed the regulatory mechanisms associated with the orphan response regulator DegU in the heat resistance of *L. monocytogenes*. The findings indicated that DegU contributes to regulating the expression of heat-shock-related genes *via* a complicated regulatory network involving the *hrcA*-*grpE*-*dnaK*-*dnaJ* operon (**Figure 5**). Many stress proteins are known to be essential for the survival of *L. monocytogenes*, both in the external environment and inside the host (Hu et al., 2007; Seifart Gomes et al., 2011; Zhang et al., 2013; Curtis et al., 2017). However, further research is needed to better understand the mechanisms underlying the signal transduction *pathways* employed by *L. monocytogenes* during environmental adaptation and host infection.

## Data Availability Statement

The original contributions presented in the study are included in the article/**Supplementary Material**. Further inquiries can be directed to the corresponding authors.

## Author Contributions

CC and HS conceived and designed the experiments. CC, FL, HJ, XX, JXu, YH, and SD performed the experiments. CC, FL, JXi, YH, LL, and XZ analyzed the data. CC, HS and FL wrote the paper. All authors contributed to the article and approved the submitted version.

## Funding

This work was supported by the National Natural Science Foundation of China (31872620, 31770040, 31972648, 32172849, and 32002358), the Fundamental Research Funds for the Provincial Universities of Zhejiang (2020KJ004), and the Natural Science Foundation of Zhejiang Province (LZ19C180001).

## Conflict of Interest

The authors declare that the research was conducted in the absence of any commercial or financial relationships that could be construed as a potential conflict of interest.

## Publisher’s Note

All claims expressed in this article are solely those of the authors and do not necessarily represent those of their affiliated organizations, or those of the publisher, the editors and the reviewers. Any product that may be evaluated in this article, or claim that may be made by its manufacturer, is not guaranteed or endorsed by the publisher.
