# Supplementary material for: The DegU Orphan Response Regulator Contributes to Heat Stress Resistance in Listeria monocytogenes
Source: Front Cell Infect Microbiol. 2021 Dec 13;11:761335. doi: 10.3389/fcimb.2021.761335 (PMC8711649; doi:10.3389/fcimb.2021.761335)
Supplement: Supplementary file 1 [file Image_1.pdf]

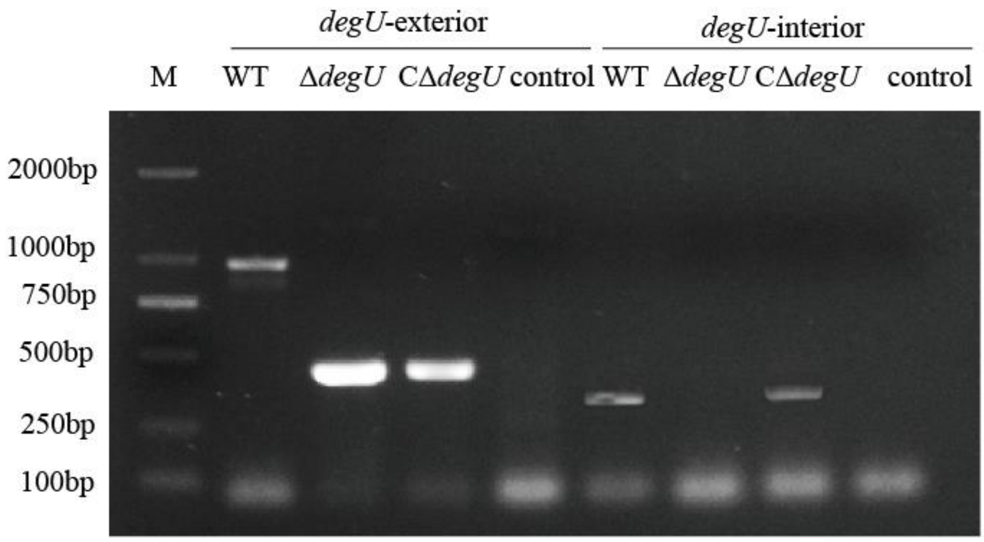

Supplementary Fig. 1. Verification of the *degU* deletion mutant and complemented strains Identification of the Δ*degU* mutant strain and the CΔ*degU* complemented strain by PCR, using PCR primer pairs exterior F/R (1012 bp for WT) and interior F/R (326 bp for WT).

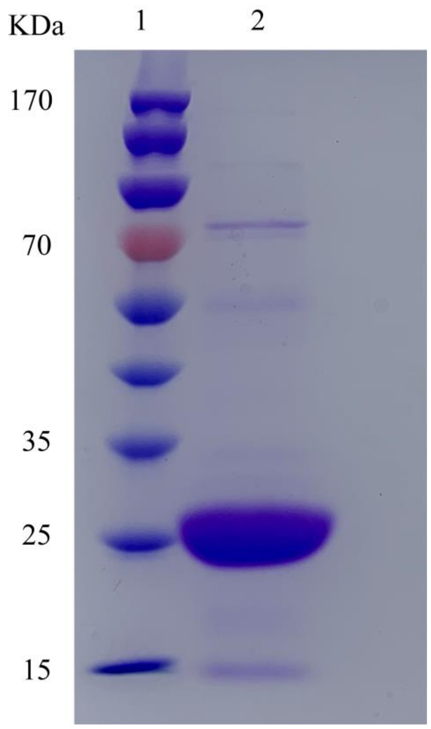

Supplementary Fig. 2. Purification of the recombinant DegU from *E.coli*. The purified DegU protein were analyzed by Coomassie staining (lane 2). The MW marker was indicated in lane 1.

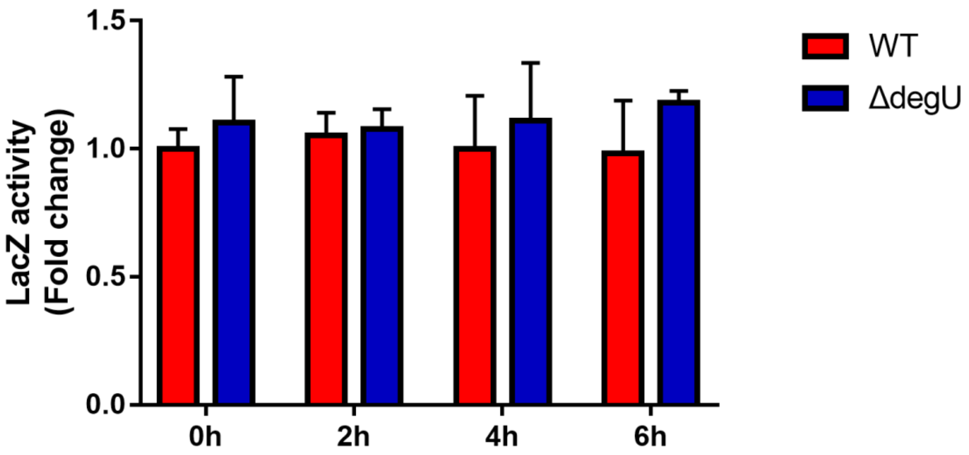

Supplementary Fig. 3 The promoter activities of *degU* in WT, Δ*degU* strains in response to heat stress.
