# Supplementary material for: The DegU Orphan Response Regulator Contributes to Heat Stress Resistance in Listeria monocytogenes
Source: Front Cell Infect Microbiol. 2021 Dec 13;11:761335. doi: 10.3389/fcimb.2021.761335 (PMC8711649; doi:10.3389/fcimb.2021.761335)
Supplement: Supplementary file 2 [file Table_1.pdf]

## Supplementary table 1. Primers used in this study

| Primer | Sequence (5′–3′) a | Use |
|---|---|---|
| *degU*-U-F | TGCTCTAGAACCAACAAGGGCTTGTGCTCCA | amplification of *degU* upstream homology arms |
| *degU* -U-R | CGAAGGTATCAAGCGAATTTTAGAGAACGGCAATCAAGCACGGCTGGGTA | |
| *degU* -X-F | TACCCAGCCGTGCTTGATTGCCGTTCTCTAAAATTCGCTTGATACCTTCG | amplification of *degU* downstream homology arms |
| *degU* -X-R | CGGGGTACCAGCTGTTCCACTAGGTTCTCCG | |
| *degU* -W-F | CTGGGGCAGGTTGAGAGGAAGT | detection exterior of *degU* mutant |
| *degU* -W-R | GGGATTATAAGGTTAAGCGGTT | |
| *degU* -N-F | TGTTAGTGCTTGCTAAATGGCG | detection interior of *degU* mutant |
| *degU* -N-R | TAAAAATATCGTAGCAAAAGTTCGC | |
| *degU-C-F* | AATTGGGGATCGGAATTCGAGCTCGGCATATAGGAGTAATTTTCTTGGG | amplification of *degU* and its promoter |
| *degU-C-R* | CGAATTCCTGCAGCCCGGGGGATCCTTAGCGAATGTATACCCAGCCGTGC | |
| *hrcA-grpE*-F | TAGCAGAATGATGGGACTCGTTGAT | amplification of *hrcA-grpE* junction used in RT-PCR |
| *hrcA-grpE*-R | TTCTTCTTGTTCGATTTCATCAGCT | |
| *grpE-dnaK* -F | GAAGCAATGAAATCACTGCGGAACT | amplification of *grpE-dnaK* junction used in RT-PCR |
| *grpE-dnaK* -R | TGCAGAGTTTGTTGTTCCTAAGTCA | |
| *dnaK-dnaJ*-F | GCAGAAGGTCAAGAAGCTCCTCAAA | amplification of *dnaK-dnaJ* junction used in RT-PCR |
| *dnaK-dnaJ*-R | CTTTTTTTATTTCGTCCGCTGAGGC | |
| *hrcA*-F | GTGCCTTCCGGTATTGTCAC | detection the transcription of *hrcA* |
| *hrcA*-R | CCGACAGGCAAATGATTCGA | |
| *DegU-F* | CGCCCATATGATGGCACTCAAAATCATGATTGTAG | amplification of *degU* for protein expression |
| *DegU-R* | CGCCCTCGAGGCGAATGTATACCCAGCCGTGCTTG | |
| *degU-F* | TTGTTGATAATGGAGGCGCG | detection the transcription of *degU* |
| *degU-R* | CCCAATTCCGCGGTTACTTT | |
| *grpE*-F | GCTAGGCTTTGCGAACGATA | detection the transcription of *grpE* |

| | | |
|---|---|---|
| *grpE*-R | GCTACGGCGGATACCTTAAC | |
| *dnaK*-F | CCTTGAATTGCAGCACCCAT | detection the transcription of *dnak* |
| *dnaK*-R | CGCCAACTCGTCAAGCATTA | |
| *dnaJ*-F | TTACCGTGAACAGTTGGCAC | detection the transcription of *dnaJ* |
| *dnaJ*-R | TCTGGTGAAGGAGAAGCTGG | |
| *rpoB*-F | ACTCTGGTGCTGCTGTAACT | detection the transcription of *rpoB* |
| *rpoB* -R | TAACAACGCGGTCTCCTTCT | |

[a] Restriction sites are underlined.
